# Effects of Pulmonary Rehabilitation on Gait Characteristics in Patients with COPD

**DOI:** 10.3390/jcm8040459

**Published:** 2019-04-05

**Authors:** Wai-Yan Liu, Kenneth Meijer, Jeannet M. Delbressine, Paul J. Willems, Emiel F. M. Wouters, Martijn A. Spruit

**Affiliations:** 1 Department of Research and Education, CIRO, 6085 NM Horn, The Netherlands; jeannetdelbressine@ciro-horn.nl (J.M.D.); e.wouters@mumc.nl (E.F.M.W.); martijnspruit@ciro-horn.nl (M.A.S.); 2 Department of Nutrition and Movement Sciences, NUTRIM School of Nutrition and Translational Research in Metabolism, Maastricht University Medical Centre+, P.O. Box 616, 6200 MD Maastricht, The Netherlands; kenneth.meijer@maastrichtuniversity.nl (K.M.); paul.willems@maastrichtuniversity.nl (P.J.W.); 3 Department of Respiratory Medicine, Maastricht University Medical Centre+, 6229 HX Maastricht, The Netherlands; 4 Department of Respiratory Medicine, NUTRIM School of Nutrition and Translational Research in Metabolism, Maastricht University Medical Centre+, 6229 ER Maastricht, The Netherlands

**Keywords:** gait variability, locomotion, lung disease, entropy, Lyapunov exponent, 3D motion analysis

## Abstract

Pulmonary rehabilitation (PR) improves lower-limb muscle function in patients with chronic obstructive pulmonary disease (COPD). However, it remains unclear whether patients improve gait characteristics, in particular stride-to-stride fluctuations that are associated with fall risks. This study aims to identify whether, and to what extent, PR affects positively gait characteristics in COPD. In this prospective observational study, 44 COPD patients (aged: 62 ± 7 years; Forced expiratory volume in 1 s 56 ± 20% predicted) performed self-paced, treadmill 6-min-walk tests (Gait Real-time Analysis Interactive Lab) before and after PR, while spatiotemporal parameters and center of mass position were recorded (100 Hz, Vicon Nexus). Standard deviation, coefficient of variation, predictability (sample entropy), and consistency in organization (local divergence exponent) were calculated. Sub-analysis was performed to identify gait differences between good and poor responders (<30 m change in a 6-min-walk distance). Patients demonstrated shorter stride times (*p* = 0.001) and improved lower-limb muscle function (*p* < 0.001) following PR. The good responders had a greater increase in stride length (*p* < 0.001) and a greater decrease in stride time (*p* < 0.001) compared to the poor responders. Current PR improved stride time in patients, while movement patterns within stride-to-stride fluctuations did not change. Training programs specifically targeting balance issues and gait function may be beneficial in improving gait characteristics in COPD.

## 1. Introduction

Walking has been reported as a problematic activity in daily life in patients with chronic obstructive pulmonary disease (COPD) [1,2]. Patients with COPD are characterized by gait alterations as compared to healthy older adults [3,4,5,6,7]. These gait alterations, in particular changes in the inherent stride-to-stride fluctuations, have been associated with non-communicable diseases [8,9] and falls in the aging population [10,11,12,13]. Moreover, stride-to-stride fluctuations may represent the capability of the locomotor system to make flexible adaptations during walking, characterized by a healthy amount of, and patterns within, these fluctuations [14]. Alterations in the amount and patterns of stride-to-stride fluctuations, whether too rigid or too random, are associated with aging, disease and falls [6,9,10,15] in many cases. In addition, gait deficits have been suggested to be an intrinsic risk factor for fall risk in COPD [16], indicating the clinical value of gait assessment in COPD. Improved insight into gait deficits in patients with COPD may direct future training programs focusing on gait function.

Gait function has been associated with muscle capacity in many older adults, suggesting a link between lower-limb muscle weakness and altered gait [17,18]. Exercise training has a beneficial effect on gait characteristics in older adults, including increased walking speed, cadence, stride length and shorter stride times [19]. Lower-limb muscle dysfunction and impaired exercise capacity are often reported in COPD [3,20]. However, pulmonary rehabilitation (PR) has proven to be an effective intervention for improving lower-limb muscle function and exercise capacity in patients with COPD [21,22,23]. Consequently, PR may have beneficial effects on gait characteristics in patients with COPD. However, whether and to what extent PR improves gait characteristics in patients with COPD is currently unknown. In addition, improvements in gait characteristics may differ between patients with COPD as their responses to PR are differential [24,25].

Within PR for COPD, the 6-min-walk test (6MWT) is used to evaluate response to treatment [26]. It has been shown that the 6-min-walk distance (6MWD) and gait characteristics can be obtained during a self-paced, treadmill 6MWT in COPD [4,27]. In the present study, we aimed to identify whether a comprehensive PR program affects gait characteristics in patients with COPD during a self-paced, treadmill 6MWT. Patients with COPD are able to walk faster during the 6MWT after PR [26]. Consequently, it is hypothesized that stride length will increase, whereas stride time and step width will decrease. In addition, we hypothesized that the amount of stride-to-stride fluctuations will decrease, and the patterns within stride-to-stride fluctuations will be less predictable and more consistent in the organization after PR, resulting in improved movement patterns. Response to PR is heterogeneous in COPD, we therefore hypothesized that good responders will demonstrate more improvements in gait characteristics as compared to poor responders.

## 2. Experimental Section

### 2.1. Study Population

This study included 44 patients with COPD who were referred for a PR program in the specialized rehabilitation center CIRO in Horn, The Netherlands. COPD diagnosis was diagnosed according to the Global Initiative for Chronic Obstructive Lung Disease (GOLD) guidelines, including post-bronchodilator spirometry (MasterScreen Body, Carefusion, Houten, The Netherlands) [28]. Patients with COPD were eligible if they were between 40–85 years of age and clinically stable evaluated by the physician. In addition, electrocardiography was conducted during pre-rehabilitation assessment and evaluated by the physician. Patients were excluded when they presented other lung diseases (e.g., asthma, lung cancer, sarcoidosis, tuberculosis or lung fibrosis), neuromuscular or orthopedic ailments affecting their gait, and/or if they used supplemental oxygen and/or walking aids. The study was approved by the Medical Research Ethics Committees United, The Netherlands (M13-1374) and registered in the Dutch Trial Registers (NTR4421). This study complied with the Declaration of Helsinki. All patients gave written informed consent prior to participating this study.

### 2.2. Study Design

This was a prospective, observational trial. Patients underwent a routine 3-day pre-rehabilitation assessment [29], in which patients performed, amongst other things, a maximal incremental cycle test during which peak work rate was determined. Patients performed a constant work rate test at 75% of the determined peak work rate and regular 6MWTs. In addition, a careful characterization of the extra-pulmonary features and comorbidities of patients with COPD were performed, which determined the application of various treatments: physical exercise training, occupational therapy, dietary counselling, psychosocial counselling, education and exacerbation management.

The pre-rehabilitation assessment was followed by a PR program in line with the American Thoracic Society/European Respiratory Society Statement on pulmonary rehabilitation [23]. PR consisted of 40 sessions that could be offered inpatient (8 weeks for 5 days/week) or outpatient (8 weeks for 3 days/week; followed by 8 weeks for 2 days/week). Physical exercise training was the cornerstone of the PR program, comprising of exercises to strengthen muscle groups in the upper and lower extremities, treadmill walking, and stationary cycling. All exercises were performed at moderate-to-high intensity to obtain an overload training stimulus, conform the Frequency, Intensity, Time, Type (FITT) principle as described by the American College of Sports Medicine [30]. The training intensity increased during the PR period, based on dyspnea and fatigue symptom scores. In addition, all patients underwent flexibility exercises, general physical exercise for lower and upper extremities, and daily supervised 30-min outdoor walks [25]. The program was implemented by an interdisciplinary team including a chest physician, respiratory nurse, dietician, occupational therapist, physiotherapist, psychologist, and social worker. 

Finally, a 2-day routine outcome assessment was conducted to evaluate the effects of the PR program for each patient. The response of PR on functional exercise capacity was assessed using the overground 6MWT [26]. Functional mobility was assessed using the Timed Up and Go test (TUG test) [31]. Body composition was assessed using dual energy X-ray absorptiometry (Prodigy, DEXAtech Benelux B.V., Ridderkerk, The Netherlands) [32]. Quadriceps muscle function (peak strength and endurance) was evaluated with a Biodex system 3 (Biodex Medical Systems Inc., New York, NY, USA). Patients performed thirty volitional maximal knee extensions at an angular velocity of 90° per second, while seated upright and with the hip joint in 90° of flexion [33].

### 2.3. Experimental Setup

Before and after PR, patients were instructed to walk on a split-belt instrumented treadmill within a virtual reality environment of a hallway (Gait Real-time Analysis Interactive Lab, GRAIL, Motek Medical B.V., The Netherlands). A Vicon motion tracking system with 10 Bonita cameras (Vicon Nexus, Oxford, UK) was used to track markers at 100 Hz [4]. These markers were placed on anatomical landmarks of the patient, according to the Human Body Model of the lower-limb (HBM1) [34]. Force plate data were sampled at 1000 Hz in synchronization with the motion capture system. Self-paced treadmill walking was enabled via a built-in controller algorithm that regulates the belt’s speed, as described in [4]. Patients performed one familiarization session. Then, two GRAIL-based 6MWTs were performed between pre-rehabilitation assessment and the first week of PR. Perceived dyspnea and fatigue (Borg scale), heart rate and pulse oxygen saturation levels were measured prior and post each 6MWT [27]. Patients performed two additional GRAIL-based 6MWTs between the last week of PR and the outcome assessment.

### 2.4. Data Analyses

The GRAIL-based 6MWT with the longest walk distance for each subject in pre and post PR was used for analyses. The first 60 s (to minimize start-up effects) and the last 15 s (to minimize deceleration effects of the treadmill) were excluded. Marker and force plate date were processed in custom MATLAB software (Mathworks Inc., Natick, MA, USA), as previously described [4]. 

All data-analysis of gait was performed on 444 consecutive steps per trial, based on the lowest number of steps of all GRAIL-based 6MWTs (range 444–684). Gait characteristics can be evaluated by computing spatiotemporal gait parameters (stride time, stride length and step width) and movement patterns of the center of mass of the subject’s body. Stride time was calculated as the time from one heel contact to the next ipsilateral heel contact. Stride length was defined as the distance between the toe marker and the ipsilateral toe marker at each heel contact in the anteroposterior direction, accounted for treadmill speed. Step width was defined as the distance between the toe markers in mediolateral direction between both feet at heel strike. The center of mass velocity (CoM_vel_) was computed using the position of the four pelvis markers.

The amount of stride-to-stride fluctuations in gait parameters was quantified by the standard deviation and coefficient of variation of spatiotemporal gait parameters (CoV = (standard deviation/mean)). Increased standard deviation and CoV values reflect a disturbed gait, as has been found in aging and disease [15]. 

The patterns within stride-to-stride fluctuations were assessed using various measures, including sample entropy and local divergence exponent (See Supplementary Materials for further description of the calculation of these measures) [8,35,36]. In short, sample entropy is a measure of predictability in the time series [36]. Sample entropy was calculated using *m* = 2, *r* = 0.2 and *n* = 444. Reduced sample entropy values reflect a more predictable gait pattern, which has been associated with a less healthy locomotor system [37]. Stride time sample entropy seemed to be a product of the parameter selection, therefore only stride length and step width sample entropy were reported. 

The CoM_vel_ was subjected to consistency analysis, in which the local divergence exponent is calculated. The method to compute this has been described elsewhere [35]. Increased local divergent exponents reflected less consistent organization of movement patterns of the patient during walking, which has been found in falls and disease [9,10,35]. Briefly, the center of mass velocity time series was reconstructed into a multidimensional space and the distances between these points were calculated as a function of time and averaged over all data points to obtain the average logarithmic rate of divergence. The slope of the divergence curve provided an estimate of the local divergence exponent, which quantifies the separation of the center of mass trajectories over time.

### 2.5. Statistics 

Data are presented as mean difference (95% Confidence Interval) unless otherwise stated. Differences in the 6MWT outcome parameters and gait characteristics between pre and post PR were compared using either paired sample T-tests or nonparametric Wilcoxon signed-rank tests. A sub-analysis was performed on good and poor responders. The minimal important difference of 30 m has been used to differentiate poor responders (<30 m improvement) and good responders (≥30 m improvement) to PR in the GRAIL-based 6MWT [26,38]. Either independent sample T-tests or nonparametric Mann–Whitney U tests were conducted to assess the differences between poor responders and good responders at baseline and the amount of changes after PR. All statistical analyses were performed in SPSS Statistics software 22.0 (International Business Machines Corporation, New York, NY, USA). The level of significance for all analyses was set at *p* ≤ 0.01.

## 3. Results

### 3.1. Baseline Subject Characteristics

Patients had mild to severe COPD (Table 1). Mean body mass index (BMI) was 26.9 ± 5.2 kg/m^2^. Patients performed the TUG test in 8.8 ± 1.3 s (Table 2). Mean quadriceps muscle strength was 71 ± 14% predicted. Patients walked 512 ± 67 m during the best overground 6MWT, corresponding to 80 ± 10% predicted, and 506 ± 75 m during the best pre-rehabilitation GRAIL-based 6MWT (Table 3). Most patients achieved their largest distance during the second 6MWT in both overground and GRAIL setting, 68% and 66%, respectively.

### 3.2. Changes after Pulmonary Rehabilitation

Fat-free mass and fat-free mass index, quadriceps muscle strength and endurance, and overground 6MWD increased significantly after PR (Table 2). The GRAIL-based 6MWD increased with 30 m after PR (95% CI (95% Confidence Interval): 14–46 m, *p* < 0.001; Table 3). Mean stride time reduced after PR (mean difference (MD): −0.02 s, 95% CI: −0.03–0.01 s, *p* = 0.001). Patients with COPD did not demonstrate significant changes in the standard deviation, CoV or predictability of spatiotemporal gait parameters; no change in consistency in the organization of center of mass patterns was found after PR.

### 3.3. Good versus Poor Responders

**Figure 1:**
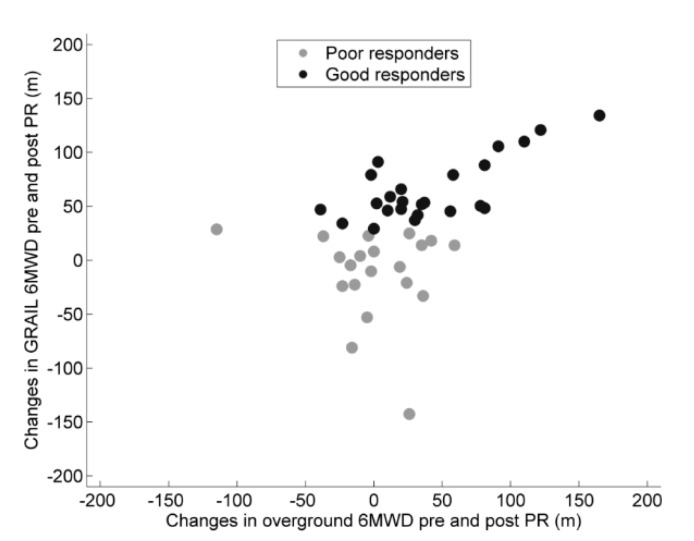
Changes in 6MWD (6-min walk distance) in good and poor responders following pulmonary rehabilitation (PR) in the overground and GRAIL condition. The good responders are depicted in black dots and the poor responders in grey dots.

Twenty-four patients (55%) achieved an improvement of 30 m or more on the GRAIL-based 6MWT, which qualified them as good responders. At baseline, no differences in 6MWT outcomes and gait characteristics were found between good and poor responders, except for mean step width, which was significantly lower in the good responders at baseline (Table 3 and Table 4). The good responders had a significantly greater improvement in 6MWD, both overground and using the GRAIL, compared to the poor responders (Figure 1). The good responders had a significantly greater increase in stride length and a greater decrease in stride time as compared to the poor responders (MD: 0.14 m, 95% CI: 0.09–0.19 m, *p* < 0.001; MD: −0.05 s, 95% CI: −0.07–0.03, *p* < 0.001, respectively, Table 5). No differences were found in the change in CoV of spatiotemporal gait parameters between the groups after PR. The change in predictability in spatiotemporal gait parameters and the change in local dynamic stability of the CoM_vel_ did not reach a statistically significant difference after PR.

## 4. Discussion

This study aimed to evaluate whether, and to what extent, a comprehensive PR program affects gait characteristics in patients with COPD during the GRAIL-based 6MWT. Patients with COPD demonstrated shorter mean stride times during the post PR GRAIL-based 6MWT. No changes in the amount of, and patterns within stride-to-stride fluctuations were found in patients following PR. In addition, good responders to PR showed improvements in mean stride time and stride length as compared to poor responders. No differences in stride-to-stride fluctuations were found between these groups after PR. The present findings suggest that a comprehensive PR program does improve walking speed in patients with COPD, whereas stride-to-stride fluctuations did not change. Research on gait impairments in COPD and identifying training modalities that improve gait function in COPD are therefore recommended.

Gait assessment may be of clinical value as gait abnormalities have been reported in patients with COPD, including reduced cadence, reduced stride length and increased double support time as compared to healthy subjects [3,4,5,6,7]. Though PR has proven to increase gait speed in patients with COPD [39], a comprehensive analysis of gait characteristics after PR is lacking. In the current study, patients with COPD improved their walk distance during the GRAIL-based 6MWT after PR, which also reached a clinically significant improvement [26]. This improvement in walking distance was achieved by a reduced mean stride time, and thus faster walking speeds. Moreover, patients with COPD tended to take longer strides contributing to an increased 6MWD. However, stride length changes were not found to be statistically significant. 

Stride-to-stride fluctuations in gait may represent the capability of the locomotor system to make flexible adaptations during walking [14]. Healthy subjects are characterized by a healthy amount of, and patterns within stride-to-stride fluctuations [40]. These stride-to-stride fluctuations are associated with fall risk and may deteriorate with aging and disease [4,6,9,10,11,41,42]. In community dwelling older adults, improvements in stride-to-stride fluctuations can be achieved following physical training [43,44]. Exercise training is a key element in PR [23] and has demonstrated to improve skeletal muscle function [22,45,46]. As skeletal muscles are important components of the locomotor system for walking, PR was hypothesized to have beneficial effects on the amount of fluctuations, predictability and consistency of movement patterns in patients with COPD. In the current study, patients with COPD did not demonstrate less variable gait characteristics, less predictable patterns of gait characteristics and increased consistency of the center of mass movement patterns. Patients did show increased fat-free mass (e.g., muscle mass), and lower-limb muscle strength and endurance. The combination of individualized strength, interval and endurance training resulted in improvements in lower-limb muscle function. However, these improvements do not necessarily translate into improvements in stride-to-stride fluctuations in patients with COPD. 

Interventions to improve walking have traditionally been multifactorial (i.e., strengthening, endurance and flexibility programs). These interventions focus on improving the physiologic capacity in body systems (i.e., musculoskeletal and cardiopulmonary systems) that contribute to walking, but do not include task specific exercises necessary to make use of the physiological capacity in body systems for walking (e.g., integrate movement and posture to improve efficiency of the physiological capacity) [47]. Therefore, task-oriented exercise training of walking could be beneficial to improve gait function, and more specifically in stride-to-stride fluctuations in COPD.

Response to PR differs in patients with COPD [25]. The current study showed that 55% of the patients were good responders in terms of improved functional exercise capacity during the GRAIL-based 6MWT (≥30 m), with an average increase in the 6MWD of 66 m after PR. The poor responders showed a decrease of 12 m during the GRAIL-based 6MWT after PR. The difference in step width at baseline was minimal between the groups and after PR this was minimal between the groups. This difference was not considered as clinically relevant. The good responders were therefore considered similar to the poor responders in terms of mean gait characteristics. Larger increase in mean walking speed for the good responders was accompanied by a shorter mean stride time and longer mean stride lengths. 

In contrast to mean spatiotemporal gait parameters between the good and poor responders, the change in the amount of, and patterns within, stride-to-stride fluctuations did not reach significance. This could be partially explained by that exercise training in PR was not specifically targeted to systems that contribute to balance and gait control. Other parameters may have contributed to the faster walking speed in the good responders. The good responders tended to start with lower dyspnea and fatigue levels compared to the poor responders during the post PR GRAIL-based 6MWT. Interestingly, some poor responders showed improvements in lower-limb muscle function. This indicates that other factors may be associated with decreased walking speed, such as higher fatigue levels prior to the post PR GRAIL-based 6MWT. Furthermore, differences in ventilator reserve may be important. However, this was not assessed in the present study.

The present study has some limitations. Externally paced treadmill walking affects gait parameters and its stride-to-stride fluctuations. The GRAIL system, however, enables self-paced treadmill walking, using a feedback-controlled algorithm to adapt treadmill speed to the user while walking. The GRAIL system has shown to increase walking speed variability [48], thereby suggesting that self-paced treadmill walking allows for a more natural control of walking speed [49]. Patients with COPD who were most severely limited in their mobility and therefore using a walking aid were not eligible for the GRAIL-based 6MWT. It would be interesting to investigate whether alterations in gait characteristics are present for patients with more severe COPD, especially given the trend that lower-limb muscle weakness is more prevalent with more severe COPD [50]. Another limitation is that the assessment of lower-limb muscle strength and endurance in our study only targeted the quadriceps muscles. Reduced muscle strength has also been observed in the distal muscles of the lower-limbs [51]. Future studies should include the assessment of distal lower-limbs to gain a better understanding of distal lower-limb muscle dysfunction and gait characteristics in patients with COPD. 

It was postulated that exercise training, and in particular, changes in lower-limb muscle function, could have positive effects on gait characteristics in COPD. This was partially confirmed by the findings of the present study, as walking speed increased. However, stride-to-stride fluctuations did not improve following PR. The current PR program was not specifically aimed to improve gait function and balance in patients with COPD. Moreover, in contrast to the study by Wang et al. [44], our study did not incorporate balance training specifically in PR. Consequently, this might explain the lack of changes in stride-to-stride fluctuations. It may be important to present exercises targeting to various systems for balance control and in different situations. We, therefore, propose to perform future research on gait and balance training in PR for patients with COPD. To date, different modalities of gait training are available, including perturbation training and dual task training [52,53], that could be used to assess the effect of gait and/or balance training on gait characteristics in COPD. In particular, improvements in stride-to-stride fluctuations will be important, as these measures are associated with increased fall risk in older adults and the high incidence of fall risk present in COPD [13].

## 5. Conclusions

This study showed that patients with COPD demonstrate shorter stride times during the GRAIL-based 6MWT after a comprehensive PR program. Though improvements in exercise capacity, body composition and quadriceps strength were found in patients with COPD following PR, stride-to-stride fluctuations did not improve. In addition, differences in mean gait characteristics were found between good and poor responders, while stride-to-stride fluctuations were not discriminative. These findings indicate that the current PR does not alter stride-to-stride fluctuations in patients with COPD. Additional training programs specifically targeting balance and gait function may be beneficial in improving gait characteristics in patients with COPD.

## Figures and Tables

**Table 1:** Patient demographic characteristics at baseline.

Variable	Pre-Pulmonary Rehabilitation (*n* = 44)
Age, years	62.2 (7.5)
Gender M/F	25/19
Weight, kg	76.5 (16.6)
Height, m	1.68 (0.18)
BMI, kg/m^2^	26.9 (5.2)
FEV_1_/FVC	0.42 (0.12)
FEV_1_% predicted	55.88 (19.73)
GOLD stage 1	6 (13.6%)
GOLD stage 2	18 (40.9%)
GOLD stage 3	17 (38.6%)
GOLD stage 4	3 (6.8%)
Never smoker	1 (2.3%)
Former smoker	41 (93.2%)
Current smoker	2 (4.6%)
Pack years	41.55 (20.6)

Data are expressed as means (standard deviation) or percentage. FEV_1_, forced expiratory volume in the first second; FVC, forced vital capacity; BMI: body mass index; GOLD: Global Initiative for Chronic Obstructive Lung Disease.

**Table 2:** Clinical outcomes before and after pulmonary rehabilitation.

		Pre-Pulmonary Rehabilitation		Post-Pulmonary Rehabilitation		
Test	Parameter	*n*	value	*n*	value	*p*-value
Body composition	Total FM, kg	43	27.3 (9.2)	43	27.1 (8.6)	0.745
	Total FFM, kg	43	47.8 (9.5) *	43	48.7 (9.7)	<0.001 ^†^
	FFMI, kg/m^2^	43	16.8 (2.4)	43	17.1 (2.4)	<0.001
Quadriceps muscle function	Peak torque, N/m	38	106 (33)	38	114 (35)	<0.001
	Peak torque, % predicted	38	71 (14)	38	77 (14)	<0.001
	Total work, J	38	1978 (625)	38	2220 (669) *	<0.001 ^†^
	Work fatigue, %	38	43 (11)	38	39 (9) *	0.048 ^†^
TUG test	Time, s	44	8.8 (1.3)	44	8.7 (1.1)	0.653
Overground 6MWT	6MWD, m	44	512 (67)	44	535 (71)	0.003
	Walking speed, m/s	44	1.42 (0.18)	44	1.5 (0.2)	0.003
	SpO_2_ rest, %	44	94 (2)	44	95 (2) *	0.760 ^†^
	SpO_2_ end, %	44	88 (6) *	44	87 (6)	0.055 ^†^
	Heart rate rest, bpm	44	85 (14)	44	80 (14)	0.014
	Heart rate end, bpm	44	115 (17)	44	117 (17)	0.318
	Dyspnea rest	44	1.2 (1.1) *	44	1.0 (1.0)	0.243 ^†^
	Dyspnea end	44	5.6 (2.0) *	44	5.1 (2.2)	0.108 ^†^
	Fatigue rest	44	1.3 (1.5) *	44	1.1 (1.2) *	0.172 ^†^
	Fatigue end	44	5.0 (2.5)	44	4.3 (2.4)	0.030

Data are expressed as means (standard deviation). FM, fat mass; FFM, fat free mass; FFMI, fat free mass index; TUG, Timed Up and Go; 6MWT, 6-min walk test; 6MWD, 6-min walk distance; SpO_2,_ pulse oxygen saturation level; bpm, beats per minute. *: indicates nonparametric value. ^†^: nonparametric Wilcoxon signed-rank test.

**Table 3:** The 6MWT outcomes and gait parameters during the GRAIL-based 6MWT before and after pulmonary rehabilitation.

			Pre-Pulmonary Rehabilitation		Post-Pulmonary Rehabilitation	
Test	Parameter	*n*	Value	*n*	value	*p*-value
6MWT	6MWD, m	44	506 (75)	44	537 (82)	<0.001
	Walking speed, m/s	44	1.43 (0.19)	44	1.48 (0.23)	0.056
	SpO_2_ rest, %	44	96 (2)	44	95 (2)	0.357
	SpO_2_ end, %	44	92 (5) *	44	93 (4) *	0.773 ^†^
	Heart rate rest, bpm	44	82 (16)	44	80 (15)	0.252
	Heart rate end, bpm	44	104 (20)	44	103 (21)	0.599
	Dyspnea rest	44	1.2 (1.1) *	44	1.0 (0.9) *	0.120 ^†^
	Dyspnea end	44	5.3 (2.2) *	44	4.6 (2.2) *	0.019 ^†^
	Fatigue rest	44	1.2 (1.2) *	44	1.1 (1.1) *	0.487 ^†^
	Fatigue end	44	4.6 (2.3)	44	4.4 (2.4)	0.494
Gait	Mean stride time, s	44	1.02 (0.08)	44	1.00 (0.08)	0.001
	Mean stride length, m	44	1.45 (0.19)	44	1.48 (0.18) *	0.037 ^†^
	Mean step width, m	44	0.18 (0.05)	44	0.18 (0.05)	0.101 ^†^
	SD stride time, s	44	0.02 (0.01)	44	0.02 (0.01)	0.599 ^†^
	SD stride length, m	44	0.05 (0.02)	44	0.04 (0.03)	0.036 ^†^
	SD step width, m	44	0.02 (0.01)	444	0.02 (0.001)	0.916 ^†^
	CoV stride time	44	0.02 (0.01) *	44	0.02 (0.01) *	0.889 ^†^
	CoV stride length	44	0.03 (0.02) *	44	0.03 (0.03) *	0.024 ^†^
	CoV step width	44	0.15 (0.07) *	44	0.15 (0.05) *	0.363 ^†^
	Sample entropy stride length	44	1.17 (0.17) *	44	1.21 (0.17) *	0.037 ^†^
	Sample entropy step width	44	1.43 (0.04) *	44	1.43 (0.05) *	0.825 ^†^
	LDE CoM_vel_-ML	44	2.83 (0.17)	44	2.77 (0.19) *	0.031 ^†^
	LDE CoM_vel_-V	44	2.78 (0.14)	44	2.79 (0.14)	0.828
	LDE CoM_vel_-AP	44	2.75 (0.15) *	44	2.70 (0.15)	0.018 ^†^

Data are expressed as means (standard deviation). 6MWT, 6-min walk test; bmp, beats per minute; SD, standard deviation; CoV, coefficient of variation; LDE, local divergence exponent; CoM_vel_, center of mass velocity; ML, mediolateral direction; V, vertical direction; AP, anteroposterior direction. *: indicates nonparametric value. ^†^: nonparametric Wilcoxon signed-rank test.

**Table 4:** Clinical outcomes of good and poor responders before and after pulmonary rehabilitation.

	Pre-Pulmonary Rehabilitation					Changes Following Pulmonary Rehabilitation				
	Good Responders		Poor Responders			Good Responders		Poor Responders		
Parameter	*n*	value	*n*	value	*p*-value	*n*	value	*n*	value	*p*-value
Age, years	24	61 (8)	20	63 (7)	0.331	24	0 (1)	20	0 (2)	0.611
Gender M, %	12	50	13	85						
BMI, kg/m^2^	24	28 (6)	20	26 (5)	0.172	24	0 (1)	20	0 (2)	0.611
FEV_1_/FVC	24	0.4 (0.1)	20	0.4 (0.1)	0.299	24	0.0 (0.1) *	20	0.0 (0.1) *	0.706
FEV_1_% predicted	24	60.2 (20.8)	20	50.7 (17.5)	0.075	24	−2.1 (4.4)	20	−2.4 (5.2)	0.852
GOLD stage 1, %	4	17	2	10		3	13	2	10	
GOLD stage 2, %	10	42	8	40		13	54	7	35	
GOLD stage 3, %	10	42	7	35		8	33	9	45	
GOLD stage 4, %	0	0	3	15		0	0	2	10	
Total FFM, kg	23	48.0 (10.9)	20	47.6 (7.8)	0.906	23	1.0 (1.6)	20	0.7 (1.2)	0.586
Peak torque, N/m	23	105 (39) *	18	111 (31)	0.358 ^†^	21	8 (12)	17	8 (12)	0.887
Peak torque, % predicted	23	72 (16)	18	72 (14)	0.916	21	7 (9)	17	5 (7)	0.544
Total work, J	23	1929 (641)	18	2055 (613)	0.526	21	279 (264)	17	196 (193)	0.289
Work fatigue, %	23	46 (11)	18	41 (11)	0.125	21	−6 (11)	17	−1 (11)	0.122
TUG test, s	18	8.6 (1.5)	16	9.0 (1.2)	0.366	18	−0.3 (1.1) *	16	0.2 (1.1)	0.427 ^†^
Overground 6MWD, m	24	520 (61)	20	503 (71) *	0.137 ^†^	24	42 (49)	20	0 (37) *	0.005 ^†^
Speed, m/s	24	1.44 (0.17)	20	1.40 (0.20) *	0.137 ^†^	24	0.12 (0.14)	20	0.00 (0.10) *	0.005 ^†^
SpO_2_ rest, %	24	94 (2)	20	95 (3)	0.546	24	1 (2)	20	−1 (3)	0.140
SpO_2_ end, %	24	88 (7) *	20	87 (5)	0.370 ^†^	24	−2 (2) *	20	0 (4)	0.067 ^†^
Heart rate rest, bpm	24	83 (15)	20	87 (12)	0.358	24	−4 (14)	20	−6 (12) *	0.777 ^†^
Heart rate end, bpm	24	113 (19)	20	116 (14)	0.632	24	6 (16)	20	−3 (14)	0.057
Dyspnea rest	24	1.0 (1.0) *	20	1.4 (1.2) *	0.289 ^†^	24	−0.5 (0.9)	20	0.2 (1.3)	0.028
Dyspnea end	24	5.9 (1.8)	20	5.3 (2.2)	0.303	24	−0.8 (1.9)	20	−0.2 (2.1)	0.306
Fatigue rest	24	1.0 (1.1) *	20	1.7 (1.8) *	0.242 ^†^	24	−0.3 (1.2)	20	−0.2 (1.8)	0.884
Fatigue end	24	4.9 (2.4)	20	5.1 (2.7)	0.774 ^†^	24	−0.7 (1.7)	20	−0.6 (2.2)	0.797

Data are expressed as means (standard deviation) or percentage. *: indicates nonparametric value. ^†^: nonparametric Mann-Whitney U Test.

**Table 5:** The 6MWT outcomes and gait parameters in good and poor responders during the GRAIL-based 6MWT before and after pulmonary rehabilitation.

		Pre-Pulmonary Rehabilitation					Changes Following Pulmonary Rehabilitation				
		Good Responders		Poor Responders			Good Responders		Poor Responders		
Test	Parameter	*n*	value	*n*	value	*p*-value	*n*	value	*n*	value	*p*-value
6MWT	6MWD, m	24	505 (60)	20	508 (91)	0.901	24	66 (29) *	20	−12 (42) *	<0.001 ^†^
	Speed, m/s	24	1.43 (0.15)	20	1.42 (0.24)	0.896	24	0.14 (0.19) *	20	−0.04 (0.15) *	<0.001 ^†^
	SpO_2_ rest, %	24	95 (2)	20	96 (2)	0.634	24	0 (2)	20	0 (2)	0.769
	SpO_2_ end, %	24	93 (6) *	20	92 (4)	0.203 ^†^	24	0 (5)	20	1 (3)	0.443
	Heart rate rest, bpm	24	84 (14)	20	82 (16)	0.858	24	−4 (12)	20	−1 (15)	0.461
	Heart rate end, bpm	24	104 (22)	20	104 (17)	0.960	24	0 (19)	20	−3 (12) *	0.550 ^†^
	Dyspnea rest	24	1.0 (0.9) *	20	1.5 (1.2) *	0.347 ^†^	24	−0.4 (1.0)	20	−0.1 (1.3)	0.356
	Dyspnea end	24	5.5 (2.1)	20	5.1 (2.4)	0.538	24	−0.7 (1.8)	20	−0.6 (2.4)	0.896
	Fatigue rest	24	1.0 (0.9) *	20	1.5 (1.4) *	0.561 ^†^	24	−0.4 (1.0)	20	0.2 (1.4)	0.128
	Fatigue end	24	4.6 (2.0)	20	4.5 (2.6)	0.910	24	−0.2 (1.8)	20	−0.2 (2.2) *	0.792 ^†^
Gait	Mean stride time, s	24	1.00 (0.06)	20	1.04 (0.09)	0.105	24	−0.05 (0.02)	20	0.01 (0.04)	<0.001 ^†^
	Mean stride length, m	24	1.43 (0.15) *	20	1.48 (0.22)	0.621	24	0.09 (0.08)	20	−0.04 (0.08) *	<0.001 ^†^
	Mean step width, m	24	0.17 (0.05)	20	0.19 (0.04) *	<0.001 ^†^	24	0.00 (0.02)	20	−0.01 (0.03)	0.136
	SD stride time, s	24	0.02 (0.01)	20	0.02 (0.01)	0.203 ^†^	24	0.00 (0.01)	20	0.00 (0.00)	0.193
	SD stride length, m	24	0.05 (0.02)	20	0.05 (0.01)	0.759 ^†^	24	0.01 (0.02)	20	0.00 (0.03)	0.109 ^†^
	SD step width, m	24	0.02 (0.01)	20	0.03 (0.01)	0.155 ^†^	24	0.00 (0.01)	20	0.00 (0.01)	0.346 ^†^
	CoV stride time	24	0.02 (0.01) *	20	0.02 (0.01) *	0.203 ^†^	24	0.00 (0.01)	20	0.00 (0.00)	0.424
	CoV stride length	24	0.04 (0.02) *	20	0.03 (0.01) *	0.588 ^†^	24	−0.01 (0.02) *	20	0.01 (0.04) *	0.017 ^†^
	CoV step width	24	0.15 (0.08) *	20	0.14 (0.05)	0.541 ^†^	24	0.00 (0.04)	20	0.00 (0.05)	0.744
	Sample entropy stride length	24	1.14 (0.19)	20	1.20 (0.13)	0.243	24	0.09 (0.19)	20	−0.01 (0.16) *	0.131 ^†^
	Sample entropy step width	24	1.43 (0.05)	20	1.43 (0.03) *	0.540 ^†^	24	0.01 (0.07)	20	−0.02 (0.06) *	0.144 ^†^
	LDE CoMvel-ML	24	2.84 (0.18)	20	2.82 (0.15)	0.734	24	−0.06 (0.16)	20	−0.06 (0.18) *	0.869 ^†^
	LDE CoMvel-V	24	2.80 (0.14)	20	2.76 (0.14)	0.417	24	0.04 (0.17)	20	−0.04 (0.14)	0.126
	LDE CoMvel-AP	24	2.76 (0.15) *	20	2.74 (0.15)	0.671 ^†^	24	−0.07 (0.13) *	20	−0.02 (0.13)	0.195 ^†^

Data are expressed as means (standard deviation). CI, confidence interval. *: indicates nonparametric value. ^†^: nonparametric Mann-Whitney U Test.

## Supplementary Materials

The following are available online at https://www.mdpi.com/2077-0383/8/4/459/s1, Supplementary Materials title: Measures to quantify the patterns within stride-to-stride fluctuations.
