# Supplementary material for: Effects of Pulmonary Rehabilitation on Gait Characteristics in Patients with COPD
Source: J Clin Med. 2019 Apr 5;8(4):459. doi: 10.3390/jcm8040459 (PMC6518247; doi:10.3390/jcm8040459)
Supplement: Supplementary file 1 [file jcm-08-00459-s001.pdf]

Appendix A. Measures to quantify the patterns within stride-to-stride fluctuations.

Sample entropy quantifies predictability and describes the predictability of gait cycles. The method to compute this has been described previously (1). A perfectly repeatable time series would reflect a sample entropy value of approximately 0, while a completely random time series would reflect a sample entropy value extending towards infinity. The relative consistency of the group averages was examined for several combinations of input parameters of the tolerance radius r and vector length m (1). The r was chosen as 0.2*standard deviation of the time series and m was chosen as 2 for this study (see Figure 1). In contrast to stride length and step width time series, stride time sample entropy seems to be a product of the parameter selection. Sample entropy values tended to direct to predictability in stride time with increasing r. Predictability of stride time itself could not be determined. Stride time sample entropy was therefore not included for statistical analysis.

Consistency in the organization of movement patterns was calculated by computing the local divergence exponent, which quantifies the exponential rate of divergence of adjacent trajectories in state space and has been described elsewhere (2). A lower local divergence exponent reflects a more consistent organization of movement patterns. An embedding dimension of 6 was used for the mediolateral, 7 for the anteroposterior and 6 for the vertical direction. The CoM_{vel} data were normalised to 100 samples in length per stride. A time delay of 10 samples was chosen as all the time series had the same frequency. The slope of the divergence curve provided an estimate of the local divergence exponent. The local divergence exponent was calculated over the CoM_{vel} in three directions. To overcome non-stationarities, velocity time series was used to estimate consistency of movement patterns (2).

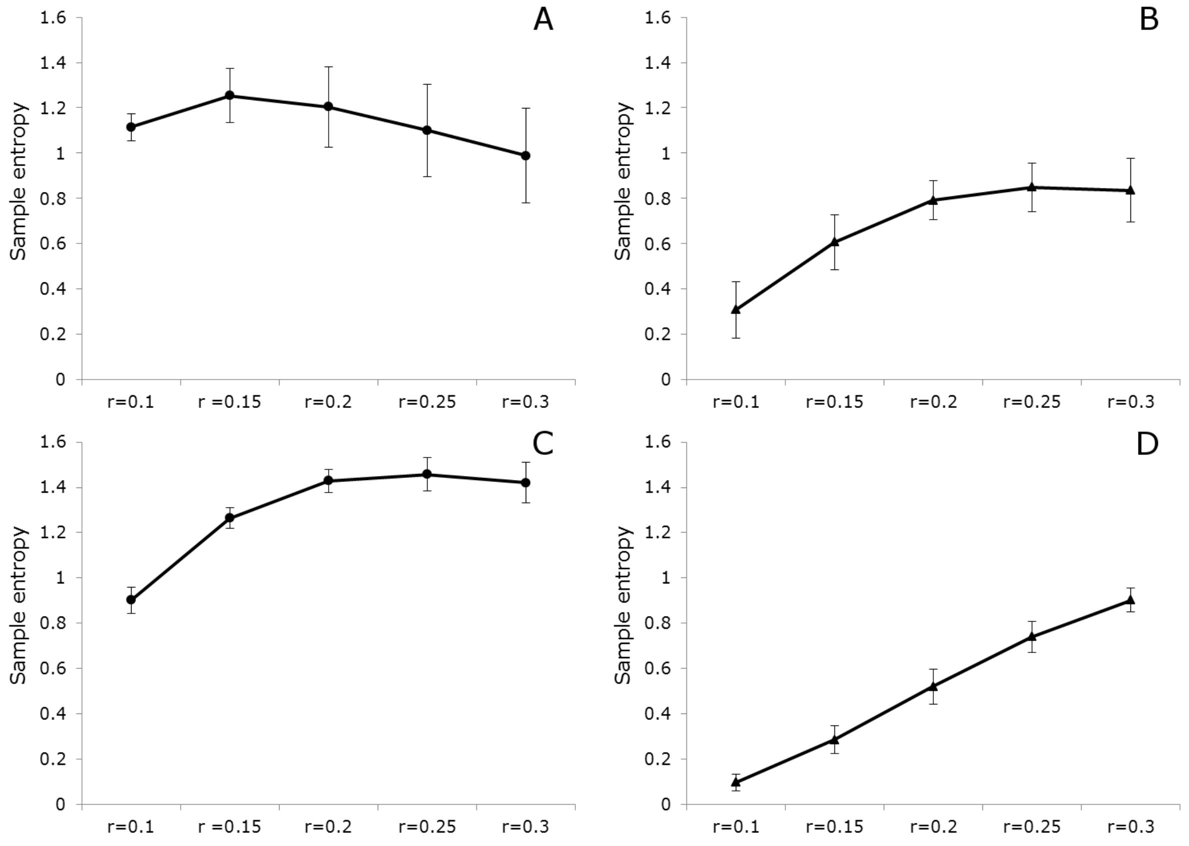

Figure 1. Sample entropy as a function of step length (A, B) and step width (C, D) for *r*=0.1-0.3, *m*=2 (left panel) and *m*=3 (right panel).

References

1. Yentes JM, Hunt N, Schmid KK, Kaipust JP, McGrath D, Stergiou N. The appropriate use of approximate entropy and sample entropy with short data sets. Annals of biomedical engineering. 2013;41(2):349-65. doi: 10.1007/s10439-012-0668-3. PubMed PMID: 23064819.

2. Bruijn SM, Meijer OG, Beek PJ, van Dieen JH. Assessing the stability of human locomotion: a review of current measures. Journal of the Royal Society, Interface. 2013;10(83):20120999. doi: 10.1098/rsif.2012.0999. PubMed PMID: 23516062; PubMed Central PMCID: PMC3645408.
